# Effects of Co Addition on the Microstructure and Properties of Elastic Cu-Ni-Si-Based Alloys for Electrical Connectors

**DOI:** 10.3390/ma14081996

**Published:** 2021-04-16

**Authors:** Zheng Wang, Jiang Li, Zhuangzhuang Fan, Yi Zhang, Songxiao Hui, Lijun Peng, Guojie Huang, Haofeng Xie, Xujun Mi

**Affiliations:** 1 State Key Laboratory of Nonferrous Metals and Processes, GRIMAT Group Co., Ltd., Beijing 100088, China; wz6252@163.com (Z.W.); huangguojie@grinm.com (G.H.); xiehaofeng@grinm.com (H.X.); mxj@grinm.com (X.M.); 2 China Academy of Space Technology, Beijing 100094, China; lijiangjyo@163.com (J.L.); fanzhuangzhuang@126.com (Z.F.); zy_pb@163.com (Y.Z.); 3 GRIMAT Engineering Institute Co., Ltd., Beijing 101407, China; 4 General Research Institute for Nonferrous Metals, Beijing 100088, China

**Keywords:** Cu-Ni-Si-based alloys, Ni/Co mass ratio, microstructure, strengthening mechanism, kinetic equation

## Abstract

The properties and microstructure evolution of quaternary Cu-Ni-Co-Si alloys with different Ni/Co mass ratios were investigated. The microstructure and morphological characteristics of the precipitates were analyzed by using electron backscatter diffraction (EBSD), transmission electron microscopy (TEM) and high-resolution transmission electron microscopy (HRTEM). The mechanical properties and conductivity of the alloys were significantly improved after the addition of Co. The grains presented an obvious growth trend with an increase in Ni/Co mass ratios, and the appropriate Co accelerated the recrystallization process. The δ-(Ni, Co)_2_Si phases of the Cu-Ni-Co-Si alloys and δ-Ni_2_Si phases of the Cu-Ni-Si alloys shared the same crystal structure and orientation relationships with the matrix, which had two variant forms: δ_1_ and δ_2_ phases. The precipitates preferential grew along with the direction of the lowest energy and eventually exhibited two different morphologies. Compared with that of the Cu-Ni-Si alloy, the volume fraction of precipitates in the alloys with Co was significantly improved, accompanied by an increase in the precipitated phase size. The addition of Co promoted the precipitation of the precipitated phase and further purified the matrix. A theoretical calculation was conducted for different strengthening mechanisms, and precipitation strengthening was the key reinforcement mechanism. Moreover, the kinetic equations of both alloys were obtained and coincided well with the experimental results.

## 1. Introduction

Recent years have witnessed more miniaturized, multifunctional and intelligent contemporary electronic products, a larger scale of integrated circuits and the increasing microthinning of lead frame materials used in integrated circuits being produced [1]. Hence, it is of great significance to develop an elastic copper alloy with high conductivity and performance while developing electrical connectors. Cu-Ni-Si-based alloys are typical age-hardened alloys with an outstanding combination of strength and electrical conductivity and serve as a key raw material for large-scale integrated circuit lead frames. Examples include C70250 and C70350, which are extensively applied to high-current connectors, such as automotive electronics and smart mobile module components [2,3].

At present, ternary Cu-Ni-Si alloys exhibit a high strength of 600–900 MPa and electrical conductivity of 35–45% International Annealed Copper Standard (%IACS), but it also means few spaces are left for developing ternary alloys [4,5]. Therefore, the method of adding trace elements is introduced to further preform the properties of alloys. For instance, the addition of Cr can improve the precipitation kinetics and accelerate the formation rate of precipitates [6,7]. Moreover, the microelements of Al [8,9], Ti [10], Zr [11], P [12] and Mg [13] have been identified to effectively enhance the physical properties of Cu-Ni-Si-based alloys. More importantly, Co shares the same effect on the precipitation process. Xiao [14] proposed that Co can prevent the spinodal decomposition and contribute to the combination among vacancies in the matrix. Krishna [15] found that the age-hardening response was strengthened, caused by the formation of the Co_2_Si phase during the aging. Zhao [16] claimed that Co plays an active role in the strengthening effect by promoting the precipitation of solute atoms and hindering the coarsening of (Cr, Co)_2_Si phases in Cu-Ni-Si-Co-Cr alloys. Our study [17] also confirmed that Co can provide additional strength by forming Co_2_Si precipitates and the existence of δ-(Ni, Co)_2_Si composite phases with different morphologies in the Cu-Ni-Co-Si alloys. Combined with previous research, the change of the Ni/Si mass ratio had a strong effect on the microstructure and properties of ternary Cu-Ni-Si alloys [18,19]. A similar method was used to study Cu-Ni-Co-Si alloys and found that different Ni/Co mass ratios also had a great impact on the microstructure and properties of the alloys [20]. Therefore, it is necessary to further explore the relationship between the internal microstructure and performance of quaternary Cu-Ni-Co-Si alloys to provide more guidance for the subsequent design and development of new alloys.

In this paper, three variants of Cu-Ni-Co-Si alloys with different Ni/Co ratios were selected, and ternary Cu-Ni-Si alloys without Co were added to study the electrical conductivity and mechanical properties. In addition, the microstructure and precipitation sequence were discussed, and the difference in the properties of the tested alloys was calculated theoretically in order to explain the relationship between the microstructure and properties of the alloys.

## 2. Experimental

The designed Cu-Ni-Co-Si alloys were prepared for the present work. The chemical compositions of the studied alloys were determined by inductively coupled plasma atomic emission spectrometry (ICP-AES), as shown in Table 1. The ingots with a dimension of 200 × 100 × 20 mm^3^ were cast in an iron die, and then the surface defects were removed. Subsequently, the cast ingots were hot-rolled to a thickness of 2 mm after multiple rapid hot rolling at 930 °C and cut into sheets with a size of 25 × 20 × 2 mm^3^. In addition, the solution treatment was performed at 900 °C for 1 h (for the NC-4 alloy) and at 1020 °C for 1 h (for the NC-1, NC-2 and NC-3 alloys), followed by quenching in water. Finally, the samples were isothermally aged at 500 °C for various times for subsequent analysis.

The microhardness test was conducted on a test machine with an indentation load of 5 kg for 15 s. Electrical conductivity was measured by an eddy current conductivity meter. The tensile specimens with a gauge length of 39.3 mm were subjected to a tensile instrument with a strain rate of 10^−3^ s^−1^. The microstructural characteristics of the different tested alloys were analyzed using electron backscatter diffraction (EBSD). The precipitates were observed with transmission electron microscopy (TEM) and high-resolution transmission electron microscopy (HRTEM) by an electron microscope. The samples were punched into a disk shape with a diameter of 3 mm and then electrolytically polished in a solution of 20% nitric acid and 80% alcohol at −40 °C.

## 3. Results

### 3.1. Physical Properties

**Figure 1:**
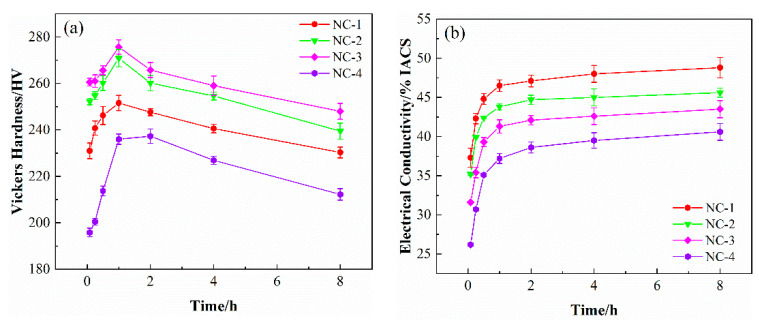
Variation curves of (**a**) hardness and (**b**) electrical conductivity of the tested alloys aged at 500 °C for different times.

Figure 1 shows the variation of the physical properties of the tested alloys aged at 500 °C for different times. The hardness and electrical conductivity of the four alloys share a similar changing trend. In the wake of the dramatic increase in hardness for the four alloys in the first 1 h, the hardness gradually decreases with the extension of the aging time, as shown in Figure 1a. The NC-1, NC-2 and NC-3 alloys peak at 1 h, while NC-4 alloy reaches the peak value after about 2 h of aging. Figure 1b presents the electrical conductivity of the alloys varying at different times. The electrical conductivity initially grows rapidly and then increases slowly, maintaining an increasing trend throughout the whole aging period. In terms of hardness, the NC-3 alloy ranks the top, followed by the NC-2, NC-1 and NC-4 alloys, while the NC-1 alloy shows the best electrical conductivity.

**Figure 2:**
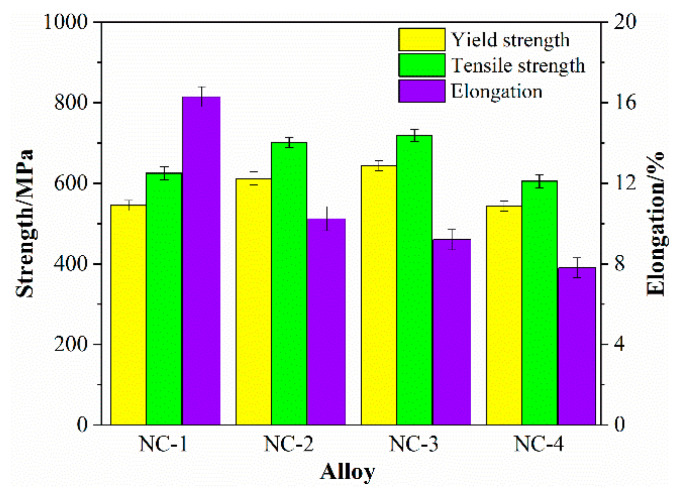
Comparison of mechanical properties of the tested alloys at the peak aging state.

Figure 2 presents a comparison of the mechanical properties of the tested alloys at the state of peak aging. The yield and tensile strengths are positively related to the hardness. Moreover, the yield strength of the four alloys exceeds 500 MPa, and the tensile strength exceeds 600 MPa. Among them, the yield and tensile strengths of the NC-3 alloy rank at the top, which are 644 and 720 MPa, respectively. When it comes to the elongation, it is negatively correlated with the strength of the alloys, which is 16.3%, 10.3%, 9.2% and 7.8%, respectively. Comparing the four alloys, the mechanical properties of the NC-3 alloy are generally better than those of the other alloys.

### 3.2. Microstructures

#### 3.2.1. EBSD Analysis of the Microscopic Structure

**Figure 3:**
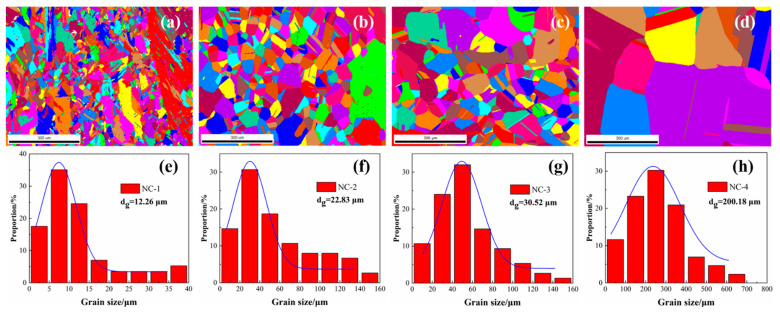
Microstructural characteristics and grain size of the tested alloys at the peak aging state. (**a**,**e**) NC-1 alloy; (**b**,**f**) NC-2 alloy; (**c**,**g**) NC-3 alloy; (**d**,**h**) NC-4 alloy.

The microstructural characteristics of the tested alloys at peak aging are presented in Figure 3. The grains present an obvious growth trend, with average grain sizes of 12 μm, 22 μm, 30 μm and 200 μm, respectively. Furthermore, the average grain size of the NC-4 alloy is almost 15 times that of the NC-1 alloy, as shown in Figure 3e,h. In general, the grains are evenly distributed inside the alloy after heat treatment, the grains of the NC-2 and NC-3 alloys are uniform and have no obvious directionality, showing a standard normal distribution in Figure 3f,g. However, the grains of the NC-1 alloy are distributed along the rolling direction in some regions, as shown in Figure 3a. Moreover, recrystallized grains are observed in the NC-1 alloy, but few are observed in others, which means the appropriate Co can accelerate the recrystallization process. The uneven grain distribution of the NC-1 alloy causes incompatible plastic deformation and local stress concentrations. Compared with that of the NC-4 alloy, the grain size of the other alloys reduces significantly, indicating that effectively Co promotes the inhibition of grain growth and the refinement of alloy microstructure.

#### 3.2.2. Microstructure Observation by TEM

**Figure 4:**
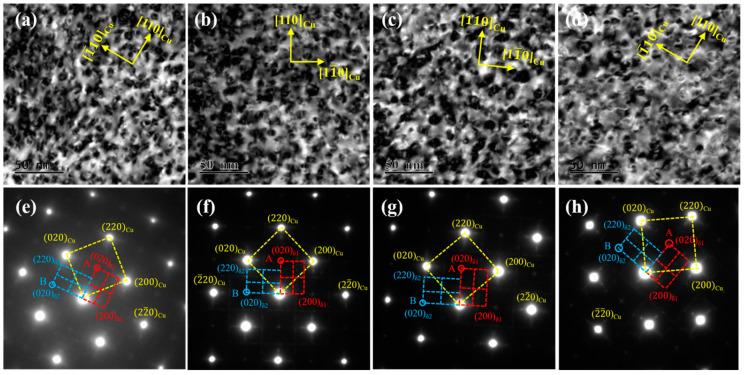
TEM images of the tested alloys at the peak aging state. (**a**–**d**) Bright-field of [001]_Cu_; (**e**–**h**) SAED of [001]_Cu_; (**a**,**e**) NC-1 alloy; (**b**,**f**) NC-2 alloy; (**c**,**g**) NC-3 alloy; (**d**,**h**) NC-4 alloy.

Figure 4 shows TEM images of the tested alloys at the peak aging state along the [001]_Cu_. direction A large number of precipitated particles are uniformly dispersed, and a small number of dislocation entanglements are distributed in the matrix. The precipitated particles in the four alloys are all bean-shaped, but there are certain differences in size, as shown in Figure 4a–d. Furthermore, the four alloys share the same selected area electron diffraction (SAED) pattern images. As illustrated by the corresponding patterns in Figure 4e,h, there are two sets of spots in addition to the matrix spots, marked as red A and blue B, respectively. Point A is located at the intersection of the centerline of the matrix spots, while Point B is close to the matrix spots. According to the calibration results, the two sets of spots are the same precipitated phase δ-(Ni, Co)_2_Si in the NC-1, NC-2 and NC-3 alloys, with an orthorhombic structure of two variant forms δ_1_ and δ_2_. Similarly, the spots in the NC-4 alloy are all the same precipitated phase δ-Ni_2_Si with two variants of δ_1_ and δ_2_, which is consistent with the previous results [4,20]. The orientation relationships (ORs) of the precipitates and matrix can be calibrated as (220)_Cu_//(020)_δ1_//(200)_δ2_ and [001]_Cu_//[100]_δ1_//[100]_δ2_.

**Figure 5:**
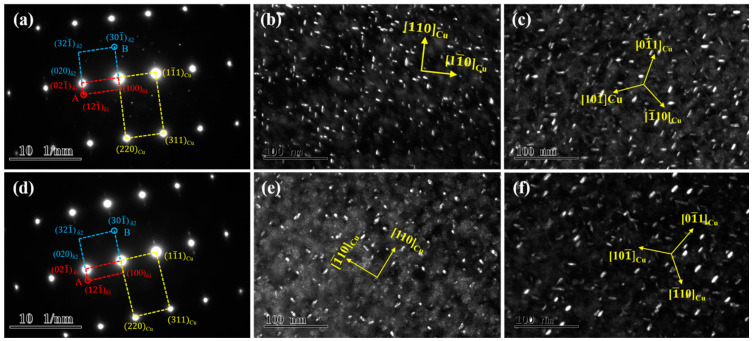
TEM images of the NC-3 and NC-4 alloys at the peak aging state. (**a**–**c**) NC-3 alloy; (**d**–**f**) NC-4 alloy; (**a**,**d**) SAED of [112]_Cu_; (**b**,**e**) dark-field of [001]_Cu_; (**c**,**f**) dark-field of [111]_Cu_.

In order to further confirm the microstructure of the precipitated phase, the NC-3 and NC-4 alloys were selected as an example, as shown in Figure 5. Figure 5c,f presents the SAED patterns of δ-(Ni, Co)_2_Si and δ-Ni_2_Si phases along the [112]_Cu_ direction are the same, which is consistent with the calibration results of the [001]_Cu_ direction. Hence, the results indicate δ-(Ni, Co)_2_Si and δ-Ni_2_Si phases share the same orthorhombic structure and ORs with the matrix. The corresponding ORs of the precipitates and matrix can be calibrated as ($1\bar{1}1$)_Cu_//($02\bar{1}$)_δ1_//(020)_δ2_, [112]_Cu_//[012]_δ1_//[010]_δ2_. According to the dark-field images of Figure 5b,e, the precipitated phases present a mutually perpendicular position relationship along the [001]_Cu_ direction. In addition, the precipitated phases are distributed at an angle of 120 degrees and all parallel to the <110>_Cu_ along the [111]_Cu_ direction, as shown in Figure 5c,f. Comparing the dark-field images of the two alloys, the addition of Co is an active player of precipitation as the number density of precipitates in the NC-3 alloy being larger than that of the NC-4 alloy.

**Figure 6:**
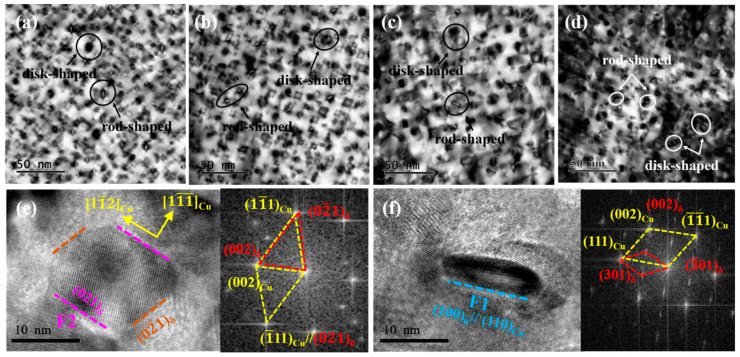
TEM and HRTEM images of the tested alloys at the peak aging state. (**a**–**d**) Bright-field of [110]_Cu_; (**e**) HRTEM image corresponding to the disk precipitate; (**f**) HRTEM image corresponding to the rod precipitate.

A large number of precipitated phases can be clearly observed from the TEM images along the [110]_Cu_ direction in Figure 6a,d, which are distributed uniformly in the matrix. Compared with the bright-field images of the four alloys, the precipitated phases share the same morphological features, except for the size, which are both rod-shaped and disk-shaped structures, respectively. The HRTEM images in Figure 6e,f show that two precipitated phases along the [110]_Cu_ direction are located on the (021)_δ_ and (301)_δ_ crystal planes, respectively. According to the corresponding Fourier transform in Figure 6e, an angle deviation of 2–3° is observed between the ($0\bar{2}1$)_δ_ and ($1\bar{1}1$)_Cu_ crystal planes, while the (021)_δ_ and ($\bar{1}11$)_Cu_ crystal planes remain parallel, and the precipitated phases are still located on the (110)_Cu_ crystal planes. This indicates that the ORs between precipitates and matrix are deflected with the aging process, resulting in the variation of growth direction.

Combined with the previous analysis, different morphologies of the precipitates were mainly ascribed to various growth directions [21]. From the Fourier transform results, the precipitate with the ORs of ($\bar{1}11$)_Cu_//(021)_δ_ and [110]_Cu_//[100]_δ_ tends to grow in a two-dimensional plane with a disk shape, while that with the ORs of (111)_Cu_//(301)_δ_ and [$\bar{1}10$]_Cu_//[010]_δ_ grows in a one-dimensional direction to form a rod shape. Caused by the diffusion during the phase transformation, the change in the lattice parameters of the precipitates results in an angle deviation of the or between the precipitates and matrix. Under the principle of energy minimum, the precipitates will preferentially grow along with the direction of the lowest energy, which exhibits two different morphologies eventually [22].

**Figure 7:**
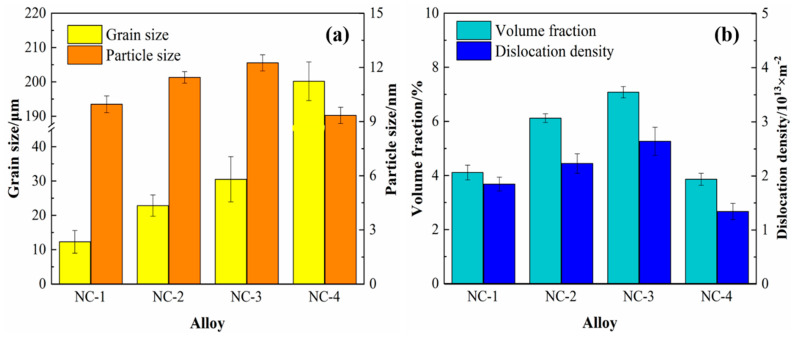
Statistics of grains and precipitates of the tested alloys at the peak aging state. (**a**) the size of grains and precipitates; (**b**) the volume fraction and dislocation density.

Figure 7 shows the statistics of the grains and precipitates of the tested alloys at the peak aging state. Compared with the four alloys in Figure 7a, the grain size of the alloys keeps growing gradually, among which the NC-4 alloy has the largest grain. In addition, the precipitated phase fluctuates within the range of 9–15 nm, and the average particle size of the NC-2 and NC-3 alloys is relatively close, 11.45 and 12.25 nm, respectively. Compared with the NC-4 alloy in Figure 7b, the volume fraction of precipitates in the NC-1, NC-2 and NC-3 alloys increases significantly. For example, the volume fraction of precipitates in the NC-3 alloy is twice that of the NC-4 alloy, 7.08% and 3.86%, respectively. Moreover, the increase in dislocation caused by rolling deformation is also calculated, and those of the four alloys are all in the order of magnitude of 10^13^. In conclusion, compared with the NC-4 alloy without Co, the volume fraction of precipitates in the alloy with Co is significantly improved, accompanied by an increase in the precipitated phase size. This indicates that the addition of Co promotes the precipitation of the precipitated phase and further purifies the matrix.

## 4. Discussion

### 4.1. Analysis of the Mechanical Properties of Cu-Ni-Co-Si Alloys

The strengthening mechanisms of Cu-Ni-Co-Si alloys are mainly divided into precipitation strengthening, solid solution strengthening, grain boundary strengthening and dislocation strengthening [23]. Under the combined action of these strengthening mechanisms, the strength of the alloy has been significantly enhanced. In order to better explain the effect of each strengthening method, quantitative calculations were carried out, respectively, and, finally, linear superposition was performed.

#### 4.1.1. Precipitation Strengthening

Precipitation strengthening is the most effective strengthening method for age-strengthened alloys. In the aging process, the supersaturated solute atoms will continuously precipitate out to form the second phase, which is uniformly distributed in the matrix. Because nanoscale second-phase particles are very hard, the dislocations can only bypass the second-phase particles during the aging process, thereby hindering the movement of dislocation [24]. Therefore, the precipitation phase is mainly caused by the Orowan bypassing mechanism to increase the yield stress to improve the alloy strength. The strength increment of the Orowan bypass mechanism in Cu-Ni-Co-Si alloys is expressed as [25]:

(1) ∆σp=0.81MGb2π(1−ϑ)12ln(dp/b)λ−dp

(2) $$\lambda =d_{p}\sqrt{\frac{3\pi }{8f_{v}}}$$

where *M* is the Taylor constant, *G* is the shear modulus of the fcc matrix, *b* is the magnitude of the Burgers vector, *ν* is Poisson’s ratio, *d_p_* is the average diameter of the precipitated phases, *λ* is the mean crystal plane spacing between precipitates and *f_v_* is the volume fraction of the precipitated phases. The value of *λ* can be estimated by Equation (2) [26]. The relevant parameters and theoretical values are listed in Table 2.

#### 4.1.2. Solid Solution Strengthening

After solid solution treatment, a large number of supersaturated solute atoms remain in the matrix. In the aging process, the supersaturated solute atoms will continuously precipitate from the matrix. However, the solute atoms cannot be completely precipitated, and the residual solute atoms in the matrix will cause the lattice distortion of the matrix. The stress field formed by the lattice distortion interacts with the stress field near the dislocations, resulting in solid solution strengthening. The solid solution strengthening increment can be expressed by the following [27]:

(3) $$∆\sigma_{s}=\sum^{\;}MG\frac{\varepsilon_{s}^{3/2}c_{x}^{1/2}}{700}$$

(4) $$\varepsilon_{s}=\left|\frac{\varepsilon_{G}}{1+\frac{1}{2}\left|\varepsilon_{G}\right|}-\beta \varepsilon_{b}\right|$$

(5) $$\varepsilon_{G}=\frac{1}{G}\frac{dG}{dc}$$

(6) $$\varepsilon_{b}=\frac{1}{a}\frac{da}{dc}$$

where $\varepsilon_{s}$ is the misfit strain caused by the lattice distortion adjacent solute, a is the lattice constant of the copper, and the value is 0.361 nm, β is 3, $c_{x}$ is the atomic concentration of the residual solute atoms and $\varepsilon_{b\;}$ and $\varepsilon_{G}$ are the correction factors for the lattice parameter and shear modulus of solid solution atoms, respectively. The detailed data are listed in Table 3, [23].

Due to the low solid solubility of Co in the copper matrix, it is assumed that all Co atoms are used to form the second-phase particles. Therefore, the solid solution strengthening of the NC-1, NC-2 and NC-3 alloys mainly comes from the residual Ni atoms in the matrix. In the subsequent calculation of conductivity, it can be found that the second-phase conversion rates of the three alloys at the peak aging state are basically the same, which are 90%, 92% and 90%, respectively. Therefore, for the convenience of calculation, the remaining Ni atom content in the three alloys was taken as the same value, and the relevant parameters and theoretical values are shown in Table 4. In addition, it can be found through the calculation that the increment of solid solution strengthening accounts for less than 5% of the total strength, so it can be ignored in the calculation.

#### 4.1.3. Grain Boundary Strengthening

In the production process, different processing methods are used to achieve the purpose of refining the grains and improving the mechanical properties of the alloy, which is called grain boundary strengthening [28]. When the grains are refined, the grain boundary area increases, and the slip bands of each grain are difficult to coordinate with each other, thus increasing the yield strength of the material. At the same time, the precipitation of the dispersed second phases can also hinder the growth of grains and further enhance the grain refinement. The grain boundary strengthening can be expressed by the following [29]:

(7) $$∆\sigma_{GB}=K_{y}d_{g}^{-1/2}$$

where *K_y_* is the Hall–Petch coefficient, reflecting the influence of surrounding particles on the flow resistance; and *d_g_* is the average diameter of grain. According to Equation (7), the relevant parameters and theoretical values are listed in Table 5.

#### 4.1.4. Dislocation Strengthening

After the material has been deformed, the dislocation density in the alloy will increase significantly so that the yield strength increases with the increment of deformation, called dislocation strengthening [30]. The increment of dislocation strengthening can be expressed by the Taylor criterion [31]:

(8) $$∆\sigma_{d}=M\alpha Gb\rho^{1/2}$$

where α is a constant, and ρ is the dislocation density, which can be obtained from the X-ray diffraction analysis. The relevant parameters and theoretical values are listed in Table 6.

#### 4.1.5. Calculated Overall Strength

**Figure 8:**
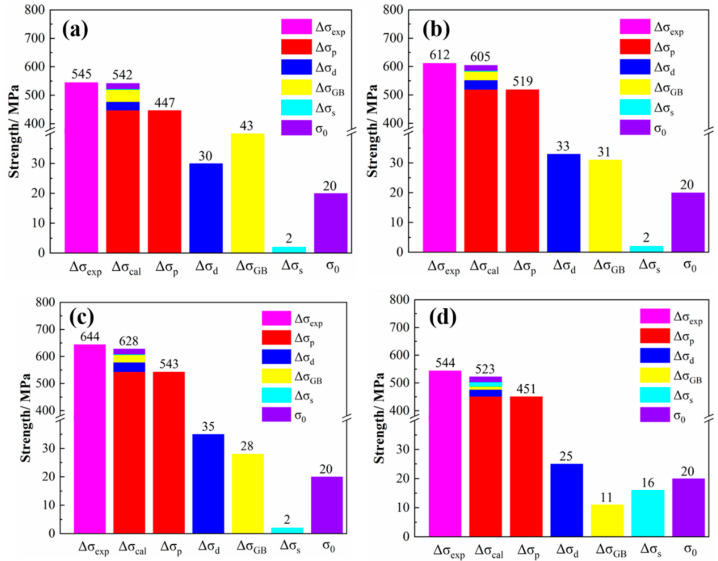
Experimental and theoretical results of yield strength of tested alloys. (**a**) NC-1 alloy; (**b**) NC-2 alloy; (**c**) NC-3 alloy; (**d**) NC-4 alloy.

The final strength of the alloy is the result of the above strengthening mechanisms, and its strength increment of $\sigma_{cal}$ can be obtained by linear summation [23,26]:

(9) $$\sigma_{cal}=\sigma_{0}+∆\sigma_{p}+∆\sigma_{GB}+∆\sigma_{s}+∆\sigma_{d}$$

where $\sigma_{0}$ represents the intrinsic lattice stress of the copper matrix. The experimental and theoretical calculation results of the yield strength are shown in Figure 8. It can be seen that the strength of the alloys is mainly derived from precipitation strengthening, and the strength increment caused by solid solution strengthening is the least. The experimental values are in good agreement with the theoretical calculation results, and the error of both is below 5%. Therefore, the above calculation model can effectively explain the strengthening effect of the alloy.

### 4.2. The Kinetics of Phase Transitions of Cu-Ni-Co-Si Alloys

Cu-Ni-Co-Si alloys will precipitate at a certain temperature and finally reach an equilibrium state. At this time, solute atoms in the matrix cannot be completely precipitated. The volume conversion rate of the precipitated phase can be defined as [32]:

(10) $$\varphi =V^{\beta }/V_{p}^{\beta }$$

where $V_{p}^{\beta }$ is the equilibrium volume of precipitate per unit volume, and$\;V^{\beta }$ is the equilibrium volume of the precipitated phase at a certain point in a unit volume. When precipitation does not occur, $V^{\beta }$ = 0, φ = 0, and the conductivity at this moment is defined as the initial conductivity$\;\varphi_{0}$. When the alloy reaches the equilibrium phase at the aging temperature,$\;V^{\beta }$ = $V_{p}^{\beta }$, φ = 1, the conductivity basically does not change when it reaches a certain value, and the corresponding conductivity is the maximum value at the aging temperature [33].

When the alloy is a low concentration solid solution, the resistivity of the solid solution can be expressed as [34]:

(11) $$\rho_{s}=\rho_{0}+a\rho$$

where $\rho_{0}$ is the resistivity of pure copper, a is the volume fraction of solute atoms and $\rho_{s}$ is the resistivity.

It can be seen from Equation (11) that the volume fraction of solute atoms is linearly related to the resistivity of copper alloy, so it can be considered that the conversion rate of the precipitated phase φ is also linearly related to the conductivity of the alloy σ.

(12) $$\sigma =\sigma_{0}+A\varphi$$

In the equilibrium state, $\sigma =\sigma_{max}$, φ=1, $A=\sigma_{max}-\sigma_{0}$, so the volume conversion rate of the precipitated phase can be obtained by the conductivity at the corresponding moment. In the aging process at 500 °C, the variation of the conversion rate of the second phase with the conductivity is shown in Table 7.

During the aging process of Cu-Ni-Co-Si alloys, the conversion rate of the precipitated phase φ and aging time *t* follow the phase transformation of the Avrami kinetic equation [35,36]:

(13) $$\varphi =1-exp\left(-bt^{n}\right)$$

where *b* is temperature constant, and *n* is the index. In order to obtain the constants *b* and *n*, the above equation can be converted to:

(14) $$1-\varphi =exp\left(-bt^{n}\right)$$

Convert the above formula and take logarithms of both sides to get:

(15) $$\mathrm{lg}\left(ln\frac{1}{1-\varphi }\right)=lgb+nlgt$$

**Figure 9:**
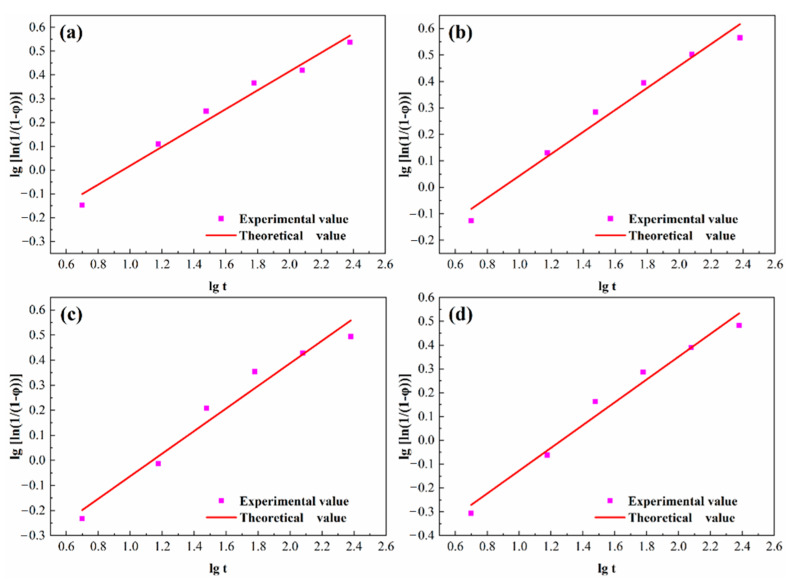
Fitting curve of precipitation phase conversion and aging time. (**a**) NC-1 alloy; (**b**) NC-2 alloy; (**c**) NC-3 alloy; (**d**) NC-4 alloy.

The result indicates the linear correlativity between $\mathrm{lg}\left(ln\frac{1}{1-\varphi }\right)$ and lgt, where the slope of the line is *n*, and the intercept is lgb. Draw the curve with $\mathrm{lg}\left(ln\frac{1}{1-\varphi }\right)\;$as the ordinate and lgt as the abscissa, and the linear fitting result is a straight line, as shown in Figure 9. On this basis, the values of *n*, *b* and the corresponding phase transition equation are obtained, as shown in Table 8.

According to Equations (12) and (13), the conductivity equation of alloys can be obtained:

(16) $$\sigma =\sigma_{0}+A\;\left[1-\exp\left(-bt^{n}\right)\right]$$

The initial value of the conductivity is $\sigma_{0}$. When the aging time is 480 min, the conductivity tends to be stable, so the conductivity at 480 min is $\sigma_{max}$. Therefore, the value of A can be obtained by using formula $A=\sigma_{max}-\sigma_{0}$. Finally, the conductivity equations of the alloys aged at 500 °C are concluded in Table 9.

**Figure 10:**
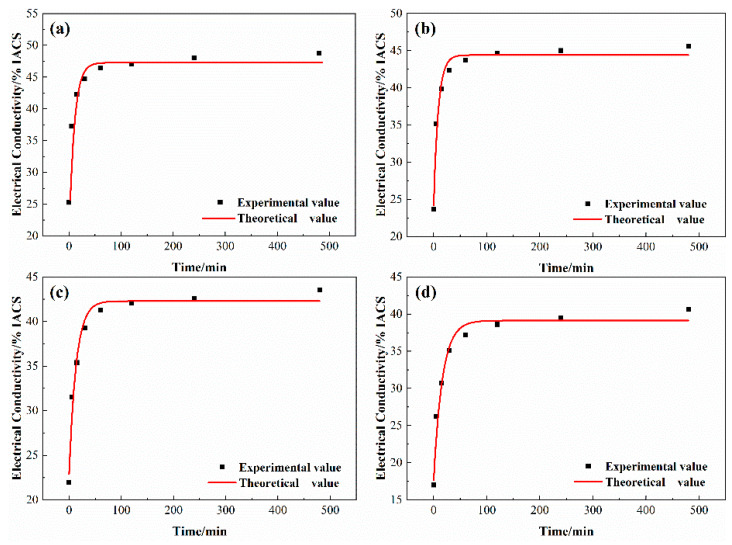
Variation of conductivity and theoretical fitting curve of alloys aged at 500 °C. (**a**) NC-1 alloy; (**b**) NC-2 alloy; (**c**) NC-3 alloy; (**d**) NC-4 alloy.

Figure 10 shows the experiment values of conductivity and the fitting curves of alloys aged at 500 °C. The comparisons between the experiment values (point, *EC*) and the theoretical values (curve, *TC*) are shown in Table 10. The error values of the calculation results are less than 5%, indicating the equations are more accurate and can better reflect the change of conductivity.

## 5. Conclusions

In this article, variations in the properties and microstructure of Cu-Ni-Co-Si alloys with different Ni/Co mass ratios were studied. The hardness and electrical conductivity of the four alloys shared a similar changing trend, and the first 1 h saw a dramatic increase in hardness and electrical conductivity. Subsequently, the hardness gradually decreased with the extension of the aging time. The electrical conductivity initially grew rapidly and then increased slowly, maintaining an increasing trend throughout the whole aging period. Compared with the NC-4 alloy without Co, the grain size of the other alloys with Co reduced significantly and effectively promoted the inhibition of grain growth and the refinement of alloy microstructure. The δ-(Ni, Co)_2_Si and δ-Ni_2_Si phases exhibited disk- and rod-shaped morphologies with an orthorhombic structure based on TEM and HRTEM characterization. The addition of Co was an active player of precipitation, with the number density and volume fraction of precipitates in Cu-Ni-Co-Si alloy being larger than that of the Cu-Ni-Si alloy, accompanied by an increase in the precipitated phase size. The theoretical calculation showed that the high strength of alloys with different Ni/Co ratios was mainly attributed to precipitation strengthening. The kinetic equations of both alloys aged at 500 °C were obtained and were consistent with the experimental data.

## Figures and Tables

**Table 1:** Testing compositions of the Cu-Ni-Co-Si alloys, wt.%.

Alloy	Cu	Ni	Co	Si	Ni/Co	(Ni + Co)/Si	Ni + Co + Si
NC-1	Bal.	0.85	2.67	0.82	0.32	4.3	4.3
NC-2	Bal.	1.84	1.64	0.82	1.12	4.2	4.3
NC-3	Bal.	2.31	1.18	0.82	1.95	4.3	4.3
NC-4	Bal.	3.48	/	0.82	N.A.	4.2	4.3

**Table 2:** Relevant parameters and theoretical values of the precipitation strengthening.

Alloy	$d_{p}\;\left(nm\right)$	$f_{v}\;(\%$)	*M*	*G* (GPa)	*b* (nm)	ϑ	$∆\sigma_{or}\;\left(MPa\right)\;$
NC-1	9.96	4.11	3.06	44	0.255	0.3	447.1
NC-2	11.45	6.12	3.06	44	0.255	0.3	518.9
NC-3	12.25	7.08	3.06	44	0.255	0.3	543.0
NC-4	9.34	3.86	3.06	44	0.255	0.3	450.8

**Table 3:** Strain energy of different solute atoms.

Solute Atoms	Lattice Parameter (nm)	Shear Modulus (GPa)	$\varepsilon_{b}$
Ni	0.352	77	−0.025
Si	0.543	65	0.504

**Table 4:** Relevant parameters and theoretical values of the solid solution strengthening.

Alloy	$c_{Ni}\;\left(\mathrm{at}.\%\right)$	$∆\sigma_{Ni}\;\left(\mathrm{MPa}\right)$	$c_{Si}\;\left(\mathrm{at}.\%\right)$	$∆\sigma_{si}\;\left(\mathrm{MPa}\right)$	$∆\sigma_{s}\;\left(\mathrm{MPa}\right)$
NC-1	0.07	2.1	-	-	2.1
NC-2	0.07	2.1	-	-	2.1
NC-3	0.07	2.1	-	-	2.1
NC-4	0.43	5.3	0.22	11.4	16.7

**Table 5:** Relevant parameters and theoretical values of the grain boundary strengthening.

Alloy	$d_{g}\;\left(\mu m\right)$	$K\;(\mathrm{MPa}\cdot \mu m^{1/2})$	$∆\sigma_{GB}\;\left(\mathrm{MPa}\right)\;$
NC-1	12	150	42.8
NC-2	23	150	31.4
NC-3	31	150	27.2
NC-4	200	150	10.6

**Table 6:** Relevant parameters and theoretical values of the dislocation strengthening.

Alloy	$\rho \;\left(m^{-2}\right)$	*M*	*G* (GPa)	*b* (nm)	α	$∆\sigma_{d}\;(\mathrm{MPa})$
NC-1	1.85 × 10^13^	3.06	44	0.255	0.2	29.5
NC-2	2.23 × 10^13^	3.06	44	0.255	0.2	32.4
NC-3	2.64 × 10^13^	3.06	44	0.255	0.2	35.3
NC-4	1.34 × 10^13^	3.06	44	0.255	0.2	25.1

**Table 7:** Conversion rate and corresponding conductivity of alloys aged at 500 °C for different times.

*t* (min)	NC-1		NC-2		NC-3		NC-4	
	*σ* (%IACS)	*φ* (%)	*σ* (%IACS)	*φ* (%)	*σ* (%IACS)	*φ* (%)	*σ* (%IACS)	*φ* (%)
0	25.3	0.0	23.7	0.0	22.0	0.0	17.0	0.00
5	37.3	51.0	35.2	52.6	31.6	44.3	26.2	39.0
15	42.3	72.4	39.9	74.1	35.4	62.2	30.7	58.0
30	44.8	82.9	42.4	85.4	39.3	80.1	35.1	76.7
60	46.5	90.2	43.8	91.7	41.3	89.6	37.2	85.6
120	47.1	92.8	44.7	95.9	42.1	93.2	38.6	91.4
240	48.0	96.8	45.0	97.5	42.6	95.6	39.5	95.2
480	48.8	100.0	45.6	100.0	43.5	100.0	40.6	100.0

**Table 8:** The phase transition equation of alloys at 500 °C.

Alloy	*n*	*b*	φ
NC-1	0.3961	0.4194	$\varphi =1-\exp\left(-0.4194t^{0.3961}\right)$
NC-2	0.4154	0.4247	$\varphi =1-\exp\left(-0.4247t^{0.4154}\right)$
NC-3	0.4504	0.3069	$\varphi =1-\exp\left(-0.3069t^{0.4504}\right)$
NC-4	0.4787	0.2478	$\varphi =1-\exp\left(-0.2478t^{0.4787}\right)$

**Table 9:** The conductivity equation of alloys aged at 500 °C.

Alloy	$\sigma_{0}\;(\%\mathrm{IACS})$	*A*	σ (%IACS)
NC-1	25.3	23.5	$\sigma =25.3+23.5[1-\exp\left(-0.4194t^{0.3961}\right)]$
NC-2	23.7	21.9	$\sigma =23.7+21.9[1-\exp\left(-0.4247t^{0.4154}\right)]$
NC-3	22.0	21.6	$\sigma =22+21.6[1-\exp\left(-0.3069t^{0.4504}\right)]$
NC-4	17.0	23.6	$\sigma =17+23.6[1-\exp\left(-0.2478t^{0.4787}\right)]$

**Table 10:** The conversion rate and corresponding conductivity of alloys aged at 500 °C for different times.

*t* (min)	NC-1		NC-2		NC-3		NC-4	
	*EC*	*TC*	*EC*	*TC*	*EC*	*TC*	*EC*	*TC*
0	25.3	25.3	23.7	23.7	22.0	22.0	17.0	17.0
5	37.3	37.2	35.2	35.0	31.6	32.1	26.2	26.8
15	42.3	41.9	39.9	39.7	35.4	35.9	30.7	30.6
30	44.8	44.1	42.4	42.3	39.3	39.4	35.1	34.9
60	46.5	46.0	43.8	43.5	41.3	41.5	37.2	37.5
120	47.1	47.3	44.7	44.6	42.1	42.1	38.6	38.6
240	48.0	48.2	45.0	45.1	42.6	42.5	39.5	39.4
480	48.8	48.4	45.6	45.4	43.5	43.2	40.6	40.1

## Data Availability

Data is contained within the article.
